# Why are nematodes so successful extremophiles?

**DOI:** 10.1080/19420889.2021.1884343

**Published:** 2021-02-19

**Authors:** Amir Sapir

**Affiliations:** Faculty of Natural Sciences, University of Haifa, Haifa, Israel

**Keywords:** Extreme environments, nematodes, adaptations, stress responses, suspended animation, oxygen, sterols

## Abstract

Extreme environments constitute the largest habitat on earth, but our understanding of life in such environments is rudimentary. The hostility of extreme environments such as the deep sea, earth’s crust, and toxic lakes limits the sampling, culturing, and studying of extremophiles, the organisms that live in these habitats. Thus, in terms of ecological research, extreme environments are the earth’s final frontier. A growing body of data suggests that nematodes are the most common animal taxon in different types of extreme settings such as the deep-subsurface and sediments in the deep sea. Notably, the reasons for the abundance of nematodes in extreme habitats remain mostly unknown. I propose that a unique combination of several characteristics of nematodes may explain, additively or synergistically, their successful adaptation to extreme habitats. Novel functional genetic and genomic approaches are expected to reveal molecular mechanisms of adaptation of nematodes to the many fascinating extreme environments on earth.

Extreme environments are characterized by a common thread of having physicochemical conditions extremely different from the norm under which most-known organisms can metabolically and biochemically operate [1,2]. This broad and strong anthropocentric criterion means that many types of habitats that differ greatly in their physicochemical properties are grouped to define extreme environments. These include, for example, the deep subsurface of the earth, deep-sea sediments and trenches, hypersaline and highly alkalic soda lakes, as well as hot and polar deserts [2]. Many types of organisms with remarkable adaptations to harsh conditions have been isolated from extreme environments. The group of extremophilic organisms consists of many species of archaea, bacteria, protists, fungi, and plants, as well as a wide range of animals such as nematodes, arthropods, tardigrade, rotifers, mollusks, and chordates [1,3]. Many extreme environments have a particular combination of several extreme conditions, e.g., some hypersaline lakes are also very alkaline and have a high concentration of arsenic [4]. In accordance with the combination of extremes, a growing body of evidence suggests that species isolated from these habitats can tolerate multiple extreme conditions. Recent studies have suggested that this cross-tolerance defines, among extremophiles, a subgroup of polyextremophiles composed of many species of microorganisms [5,6] and the members of only one phylum of animals – the tardigrades [6].

Although not presently defined as polyextremophiles, studies of species richness and abundance of individuals have highlighted nematodes as the most abundant animal taxon in samples collected from many types of extreme environments (Figure 1). For example, the analysis of samples collected from deep-sea sediments, which cover more than 65% of the earth’s surface, revealed that among the meiofauna (benthos with a body size between 50 and 1000 µM) nematodes are the most abundant metazoan taxon [7]. Moreover, the dominance of nematodes increases with water depth by up to >90% [8] suggesting a positive correlation between extreme conditions and nematode abundance. Nematodes are the dominant animal phylum in many additional extreme environments including deep-subsurface habitats [9], hot or polar deserts [10], and parts of the intestine – an anaerobic environment found inside every one of us. Nematodes are estimated to be the most common parasites in the human alimentary system inhabiting the intestines of more than 1.2 billion people worldwide [11,12].

**Figure 1.**
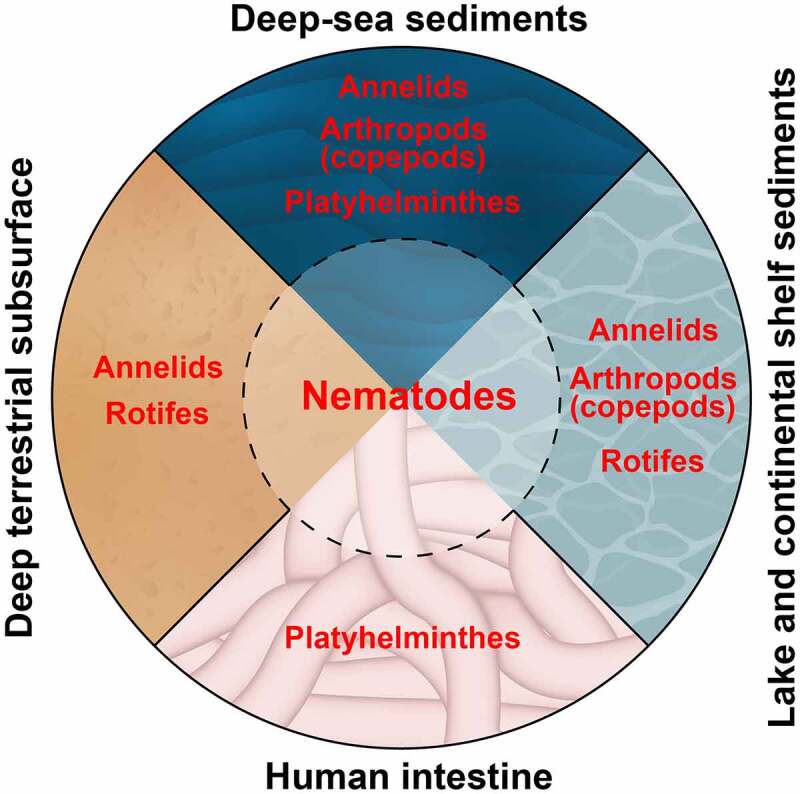
Nematodes dominate different types of extreme environments

Phyla of the most abundant species in selected extreme habitats are presented. In many of these habitats, Nematoda is the dominant phylum among the meiofauna fraction or even among all animals. Data about deep-sea habitats as well as lake and continental shelf sediments are from [14]. The information about the deep terrestrial subsurface is from [9] and about the human intestine is from [11].

I wish to propose a unique combination of five major characteristics additively or synergistically that make nematodes particularly successful in many types of extreme environments. The five characteristics are: **I**. Cylindrical body with no major appendages. This structural attribute is advantageous for animals living in granular habitats such as terrestrial or marine sediments or the intestine of animals. **II**. Adaptation to low concentrations of oxygen. Nematodes can thrive in oxygen concentrations as low as 1.44 µM [13] – more than thousand times lower than atmospheric oxygen at sea level, and accordingly are the most common animal taxon in many environments with low oxygen concentration [14]. **III**. Minimal dietary requirements for some free-living nematodes met by bacteria and sterols. Bacteria live in almost every environment on earth including many extreme habitats [15]; thus, the bacterivory of many free-living nematodes is a major advantage for occupying these niches. Sterol synthesis requires oxygen [16]; thus, mechanisms that support sterol auxotrophy in nematodes [17,18] concomitant with an adaptation to thrive in concentrations of dietary sterols as low as 30 ng/ml [19] probably represent another adaptation of nematodes to thrive in anaerobic environments. **IV**. Nematodes have various strategies for suspended animation, which enable them to survive through periods of unfavorable conditions [1,20]. The best-documented demonstration of an animal surviving intracellular freezing as a form of suspended animation were laboratory experiments with the Antarctic nematode *Panagrolaimus davidi* [21]. Supporting this finding, live nematodes were recovered from samples collected from Siberian permafrost formed 30,000–40,000 years ago [22], demonstrating a remarkable adaptation of nematodes in the wild to wait, possibly indefinitely, for favorable conditions. **V**. Preadaptation and cross-tolerance. A growing body of evidence suggests that a single defense mechanism, i.e., detoxification, can protect nematodes from different types of stress [23]. Thus, the adaptation of nematodes to one extreme habitat can confer resilience to other extreme environments through only a handful of defense mechanisms.

Notably, None of the five characteristics are unique to nematodes. Moreover, these characteristics likely contribute to the adaptation of animals from other taxa to extreme environments (Figure 1). Cylindrical body with no major appendages shared by nematodes, annelids, platyhelminths, and rotifers likely contributes to their abundance in granular habitats like deep-sea sediments. However, copepods that have long anterior appendages are also very abundant in deep-sea sediments [14]. In addition to nematodes, rotifers and copepods were reported to enter suspended animation [24,25] which can enable their adaptation to specific extreme habitats. Moreover, in samples collected from deep anoxic sediments, as well as polar ecosystems and melting ice, loriciferans, and rotifers, respectively, are more abundant than nematodes [14,26]. Consistent with these findings, some extremophiles exhibit a very high tolerance to specific extreme conditions in which nematodes may not survive. Taking pH as an example, ephydrid flies can survive in a pH as low as 2 units and rotifers in pH as high as 10.5 units [1]. Based on the notion that no single characteristic can explain why nematodes are such successful extremophiles, I wish to suggest a model for inhabitation in which the five characteristics described above predispose nematodes to be polyextremophilic organisms. Although nematodes are very abundant in moderate environments, the combination of these five characteristics this combination likely supports the pre-adaptation and cross-tolerance of nematodes to habitats of intermediate conditions at the margins of extreme environments. From this pool of preadapted species, specific nematodes undergo further adaptation that enables the colonization and, in many cases, domination of specific extreme environments. Supporting this model, in samples collected from Mono Lake, CA, a decrease in species richness was reported in the most extreme habitats in comparison to more moderate settings at the lake shorts [27].

So far, efforts to reveal the mechanisms of adaptation of extremophilic nematodes to their environments have been hindered by two major obstacles. i) The particular and hostile conditions of extreme environments limit the sampling and long-term laboratory culturing of extremophiles. Establishing a stable culture in the laboratory is usually the first critical step for the study, in a controlled experimental system, of the physiological basis of extremophiles adaptation. In recent years, however, novel approaches for replicating the conditions of extreme habitats in the laboratory have been developed and have unveiled different physiological aspects of the adaptation of nematodes to particular extreme settings [21,27]. ii) There has been a lack of tools for efficient functional genetics and genomics in extremophiles that are, by nature, non-model organisms. The recent development of genome sequencing, RNA-sequencing, RNA interference, and CRISPR-Cas9 technologies opens a new and exciting avenue toward the study of the molecular basis of adaptation of nematodes and other animals to extreme habitats. The long-term culture of extremophiles in the laboratory combined with functional genomics is expected to deepen our mechanistic understanding of the unique combination that makes nematodes so successful as extremophiles.

## References

[1] Rothschild LJ, Mancinelli RL. Life in extreme environments. Nature. 2001;409:1092–1101. 11234023 10.1038/35059215

[2] Merino N, Aronson HS, Bojanova DP, et al. Living at the extremes: extremophiles and the limits of life in a planetary context. Front Microbiol. 2019;10:780. 31037068 10.3389/fmicb.2019.00780 PMC6476344

[3] Boothby TC. Mechanisms and evolution of resistance to environmental extremes in animals. Evodevo *EvoDevo instead of Evodevo*. 2019;10:30. 10.1186/s13227-019-0143-4 PMC6862762 31827759

[4] Oremland RS, Stolz JF, Hollibaugh JT. The microbial arsenic cycle in Mono Lake, California. FEMS Microbiol Ecol. 2004;48:15–27. 19712427 10.1016/j.femsec.2003.12.016

[5] Capece M, Clark E, Saleh J, et al. Polyextremophiles and the constraints for terrestrial habitability. In: Seckbach J, Oren A, Stan-Lotter H editors. Polyextremophiles. Cellular Origin, Life in Extreme Habitats and Astrobiology. Dordrecht: Springer. 2013;27:3–59.

[6] Seckbach J, Rampelotto P. Polyextremophiles. in: Bakermans C. editors. Microbial Evolution under Extreme Conditions. DeGruyter. 2015;8:153–170

[7] Danovaro R, Bianchelli S, Gambi C, et al. α-, β-, γ-, δ- and ε-diversity of deep-sea nematodes in canyons and open slopes of Northeast Atlantic and Mediterranean margins. Mar Ecol Prog Ser. 2009;396:197–209.

[8] Danovaro R, Gambi. C, Della Crocem N. Meiofauna hotspot in the Atacama Trench, eastern South Pacific Ocean. Deep Sea Res Part I Oceanograp Res Papers. 2002;49:843–857.

[9] Borgonie G, Linage-Alvarez B, Ojo AO, et al. Eukaryotic opportunists dominate the deep-subsurface biosphere in South Africa. Nat Commun. 2015;6:8952. 26597082 10.1038/ncomms9952 PMC4673884

[10] Andrássy I. Nematodes in the sixth continent. J nematode morphol syst. 1998;1:107-108.

[11] de Silva NR, Brooker S, Hotez PJ, et al. Soil-transmitted helminth infections: updating the global picture. Trends Parasitol. 2003;19:547–551. 14642761 10.1016/j.pt.2003.10.002

[12] Epe C. Intestinal nematodes: biology and control. Vet Clin North Am Small Anim Pract. 2009;39(1091–1107):vi–vii. 10.1016/j.cvsm.2009.07.002 19932365

[13] Urkmez D, Brennan ML, Sezgin M, et al. A brief look at the free-living Nematoda of the oxic/anoxic interface with a new genus record (Trefusia) for the Black Sea. Oceanolog Hydrobiol Stud. 2015;44:539–551.

[14] Zeppilli D, Leduc D, Fontanier C, et al. Characteristics of meiofauna in extreme marine ecosystems: a review. Mar Biodivers. 2018;48:35–71.

[15] Flemming HC, Wuertz S. Bacteria and archaea on Earth and their abundance in biofilms. Nat Rev Microbiol. 2019;17:247–260. 30760902 10.1038/s41579-019-0158-9

[16] Brown AJ, Galea AM. Cholesterol as an evolutionary response to living with oxygen. Evolution. 2010;64:2179–2183. 20394667 10.1111/j.1558-5646.2010.01011.x

[17] Shamsuzzama L, Trabelcy R, Langier Goncalves B, et al. Metabolic reconfiguration in C. elegans suggests a pathway for widespread sterol auxotrophy in the animal kingdom. Curr Biol. 2020;30:3031–3038 e3037. 32559444 10.1016/j.cub.2020.05.070

[18] Capell-Hattam IM, Brown AJ. Sterol evolution: cholesterol synthesis in animals is less a required trait than an acquired taste. Curr Biol. 2020;30:R886–R888. 32750350 10.1016/j.cub.2020.06.007

[19] Merris M, Wadsworth WG, Khamrai U, et al. Sterol effects and sites of sterol accumulation in Caenorhabditis elegans: developmental requirement for 4alpha-methyl sterols. J Lipid Res. 2003;44:172–181. 12518036 10.1194/jlr.m200323-jlr200

[20] Antebi A. Steroid regulation of C. elegans diapause, developmental timing, and longevity. Curr Top Dev Biol. 2013;105:181–212. 23962843 10.1016/B978-0-12-396968-2.00007-5

[21] Wharton D, Ferns D. Survival of intracellular freezing by the Antarctic nematode Panagrolaimus davidi. J Exp Biol. 1995;198:1381–1387. 9319273 10.1242/jeb.198.6.1381

[22] Shatilovich AV, Tchesunov AV, Neretina TV, et al. Viable nematodes from late Pleistocene permafrost of the Kolyma River Lowland. Dokl Biol Sci. 2018;480:100–102. 30009350 10.1134/S0012496618030079

[23] Zhao YL, Wang DY. Formation and regulation of adaptive response in nematode Caenorhabditis elegans. Oxid Med Cell Longev. 2012;2012:564093. 22997543 10.1155/2012/564093 PMC3446806

[24] Tunnacliffe A, Lapinski J, McGee B. A putative LEA protein, but no trehalose, is present in anhydrobiotic bdelloid rotifers. Hydrobiologia. 2005;546:315–321.

[25] Lenz PH, Roncalli V. Diapause within the context of life-history strategies in calanid copepods (Calanoida: crustacea). Biol Bull. 2019;237:170–179. 31714852 10.1086/705160

[26] Danovaro R, Dell’Anno A, Pusceddu A, et al. The first metazoa living in permanently anoxic conditions. BMC Biol. 2010;8:30. 20370908 10.1186/1741-7007-8-30 PMC2907586

[27] Shih PY, Lee JS, Shinya R, et al. Newly identified nematodes from Mono Lake exhibit extreme arsenic resistance. Curr Biol. 2019;29:3339–3344 e3334. 31564490 10.1016/j.cub.2019.08.024

